# EpiGIS pro: an AI-powered geospatial intelligence platform for integrated disease surveillance and predictive analytics

**DOI:** 10.1186/s12942-026-00479-1

**Published:** 2026-06-16

**Authors:** Chenxi Guo, Peter Scott

**Affiliations:** Research Computing Centre, The University of Queensland, Brisbane, QLD Australia

**Keywords:** Disease surveillance, Geospatial information system, Machine learning, Time-series forecasting, Large language model, Epidemiological intelligence, Influenza, PostGIS

## Abstract

**Background:**

Global infectious disease surveillance requires timely integration of heterogeneous data sources. Existing platforms typically address only one dimension, leaving cross-domain correlations unexplored. This study presents EpiGIS Pro, an AI-powered geospatial platform that consolidates automated disease event extraction, environmental monitoring, mobility analysis, and predictive analytics within a single web-based framework, providing a shared data infrastructure for future cross-domain modeling.

**Methods:**

EpiGIS Pro employs a modular multi-application architecture built on Django REST Framework with PostGIS spatial extensions. Disease intelligence is automated from six source categories using Claude AI (Anthropic) for structured extraction with standardized epidemiological metadata. Environmental data, traffic congestion indicators, and international flight route data are ingested via scheduled Celery Beat tasks. Machine learning modules include Prophet-based time-series forecasting with multiplicative seasonality for WHO FluNet data, seasonal Z-score anomaly detection, and effective reproduction number (R_t) estimation. Semantic search is enabled through Voyage AI embeddings stored in pgvector-indexed PostgreSQL. A systematic evaluation framework covers six analytical dimensions across 9 countries spanning 6 years of data.

**Results:**

The platform integrates 327,000 + records across six data domains. Claude AI extraction processed 353 disease event articles from 56 countries with 92.4% success and 100% completeness for disease type, severity, and priority fields. Using 2,868 WHO FluNet records across 9 countries (2019–2026), seasonality analysis correctly identified hemisphere-concordant peak timing in all 7 evaluated countries. Prophet multiplicative forecasting reduced RMSE by 21–29% compared to the seasonal naive baseline for the United States, and consistently outperformed log-transformed Prophet across all countries and horizons. R_t estimation demonstrated epidemiologically consistent patterns, with onset-period R_t exceeding trough-period R_t in Australia (1.27 vs. 1.09) and Japan (1.80 vs. 1.08). Cross-border risk assessment computed 4,828 flight corridor risk scores by linking disease activity with 633 international aviation routes.

**Conclusions:**

EpiGIS Pro demonstrates that consolidating AI-driven event extraction, multi-source environmental and mobility data, and geospatial analytics within a unified platform enhances situational awareness for global disease surveillance. The modular architecture and reproducible evaluation framework position EpiGIS Pro as both a practical surveillance tool and a research testbed for computational epidemiology.

**Supplementary Information:**

The online version contains supplementary material available at https://doi.org/10.1186/s12942-026-00479-1.

## Background

The emergence of novel pathogens and the accelerating pace of global travel have underscored the need for integrated disease surveillance systems capable of synthesizing diverse data streams in near real-time [1, 2]. Traditional public health surveillance relies predominantly on laboratory-confirmed case reports that often lag behind the actual epidemic trajectory by days to weeks [3]. Event-based surveillance (EBS), which mines unstructured digital sources such as news articles, social media, and official health agency reports, has gained recognition as a complementary approach for early warning [4, 5].

Several platforms have advanced the state of event-based disease surveillance. ProMED-mail, one of the earliest systems, relies on expert moderation of user-submitted reports [6]. HealthMap pioneered automated aggregation of online news sources with geographic visualization [7]. The Epidemic Intelligence from Open Sources (EIOS) initiative by WHO provides a collaborative framework for monitoring public health threats [8]. More recently, BioBERT and other domain-specific language models have been applied to biomedical named entity recognition tasks relevant to disease extraction [9].

Despite these advances, existing platforms exhibit several limitations. First, most rely on rule-based or classical NLP methods for information extraction, which struggle with the diversity of disease reporting formats across languages and sources [10]. Second, environmental determinants of disease transmission—temperature, humidity, rainfall—are rarely co-located within surveillance platforms alongside disease data, despite strong evidence for climate-disease correlations [11, 12]. Third, human mobility data, including aviation passenger flows and urban transport congestion, are seldom available within the same analytical environment as disease surveillance data, even though international air travel is recognized as the primary vector for cross-border pathogen dissemination [13, 14]. Fourth, although geographic information systems (GIS) and spatial analysis methods—including cluster detection, proximity analysis, and disease mapping—have become central to spatial epidemiology [15, 16], and geospatial approaches are increasingly applied to infectious disease surveillance [17], few platforms offer an integrated analytical pipeline that combines these spatial modalities with AI-driven extraction, environmental monitoring, and predictive analytics within a unified framework [18].

The emergence of large language models (LLMs) presents new opportunities for disease surveillance. LLMs such as GPT-4 [19] and Claude [20] have demonstrated remarkable capabilities in structured information extraction from unstructured text, potentially surpassing traditional NLP pipelines in flexibility and accuracy for low-resource and multilingual settings [21]. However, the systematic application of LLMs to disease event extraction with standardized epidemiological metadata schemas remains underexplored.

Furthermore, the integration of surveillance data with predictive analytics—particularly for influenza, which exhibits well-characterized seasonal patterns amenable to time-series modeling—offers opportunities for anticipatory public health response [22, 23]. The WHO Global Influenza Surveillance and Response System (GISRS) provides standardized influenza data through the FluNet platform, yet tools for accessible forecasting and anomaly detection on these data remain limited outside specialized research groups [24].

This paper presents EpiGIS Pro, an AI-powered geospatial intelligence platform designed to address these gaps. The platform makes four primary contributions:

1. **LLM-based disease event extraction**: Automated structured extraction from heterogeneous news sources using Claude AI with a standardized epidemiological metadata schema, achieving 92.4% processing success across six source categories and 56 countries.
2. **Multi-source environmental and mobility integration**: Unified ingestion of weather data (8 cities, sub-hourly resolution), traffic congestion indicators (45 urban road segments), and international aviation routes (633 routes, 28 airports) with automated risk scoring.
3. **Integrated predictive analytics**: Prophet-based time-series forecasting with multiplicative seasonality on WHO FluNet data, seasonal Z-score anomaly detection, effective reproduction number (R_t) estimation, and PostGIS-enabled spatial analysis—all accessible through a single web interface.
4. Reproducible evaluation framework: A systematic evaluation covering six analytical dimensions—(1) data coverage, (2) AI extraction quality, (3) seasonality validation, (4) forecast accuracy, (5) anomaly detection performance, and (6) effective reproduction number (R_t) estimation consistency—across 9 countries and 327,000 + records, with multi-model cross-validation against a seasonal naive baseline, enabling transparent performance assessment.

## Methods

### System architecture

**Fig. 1:**
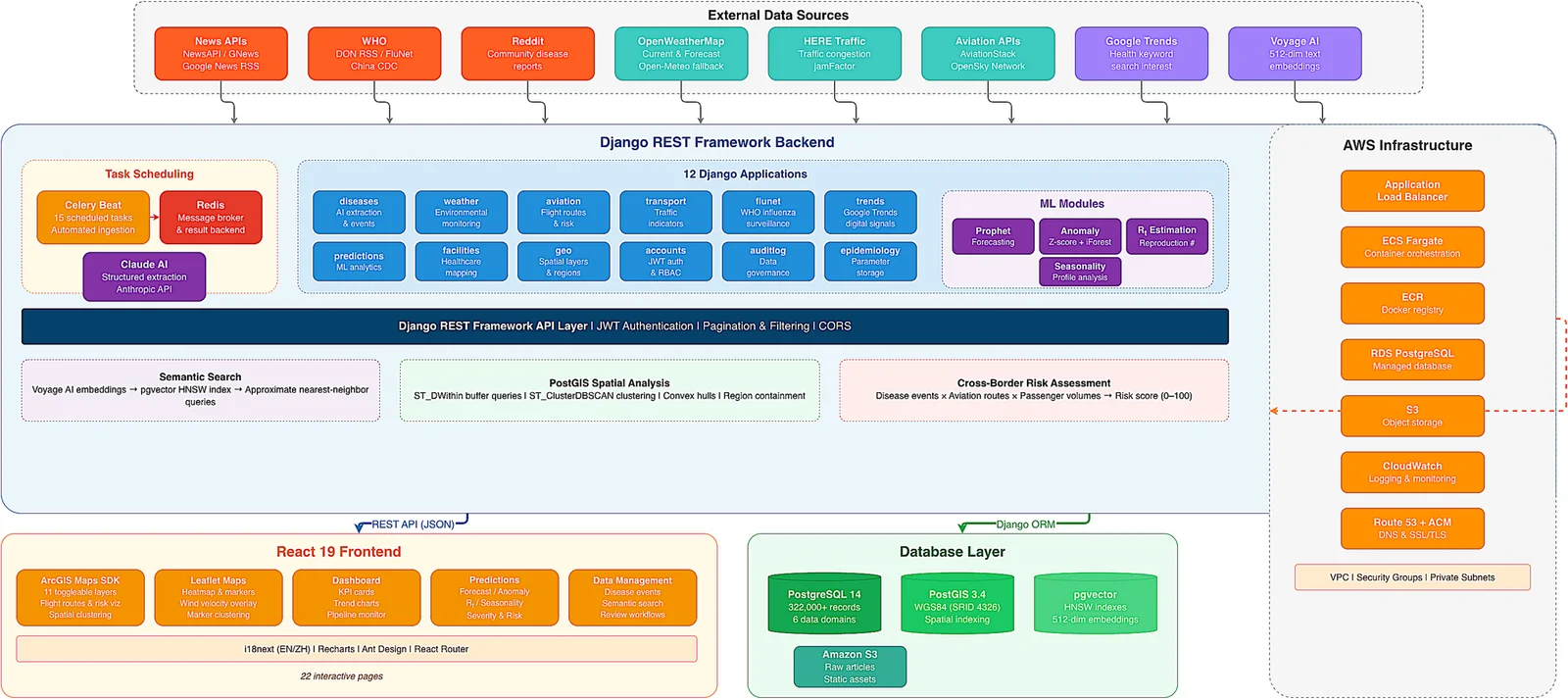
System architecture of EpiGIS Pro showing the three-tier design: React frontend with ArcGIS/Leaflet visualization, Django REST Framework backend with 12 domain applications and Celery Beat task scheduler, and PostgreSQL/PostGIS database with pgvector extension. External data sources are shown at the top, and the AWS deployment infrastructure on the right

EpiGIS Pro follows a three-tier architecture consisting of a React-based frontend, a Django REST Framework backend, and a PostgreSQL/PostGIS database layer (Fig. 1). The system is containerized using Docker and deployed on Amazon Web Services (AWS) Elastic Container Service (ECS). Key software versions are: Python 3.13, Django 6.0, React 19.2, PostgreSQL 16 with PostGIS 3.4, pgvector 0.3, Prophet 1.3, Celery 5.6 [32], ArcGIS Maps SDK 4.34, and Leaflet via react-leaflet 5.0.

#### Backend

The backend comprises 12 Django applications spanning disease surveillance, environmental monitoring, aviation and transport mobility, influenza surveillance, predictive analytics, healthcare facility mapping, and spatial analysis. All location-based models use PostGIS geometry fields, enabling spatial indexing and queries including buffering, region containment, and nearest-neighbor search. Automated data ingestion is managed by a distributed task scheduler; Table 1 summarizes the scheduled tasks and their frequencies.

**Table 1:** Automated data ingestion schedule via Celery Beat

Task	Schedule	Data Source	Purpose
fetch_news_and_extract	Twice daily (06:00, 18:00)	NewsAPI.org	Disease event extraction via Claude AI
fetch_who_news_and_extract	Twice daily (06:05, 18:05)	WHO DON RSS	WHO outbreak intelligence extraction
fetch_reddit_disease_posts	Twice daily (06:10, 18:10)	Reddit	Community-reported disease signals
fetch_gnews_and_extract	Twice daily (06:15, 18:15)	GNews API	Global news disease extraction
fetch_google_news_rss	Twice daily (06:20, 18:20)	Google News RSS	RSS-based disease monitoring
fetch_china_cdc_data	Weekly (Monday 04:00)	China CDC	National surveillance data
fetch_openweathermap_current	Every 30 min	OpenWeatherMap/Open-Meteo	Current weather conditions
fetch_wind_grid_data	Every 6 h	OpenWeatherMap	Wind grid data
fetch_traffic_data	Every 15 min	HERE Traffic API	Traffic congestion indicators
fetch_flights_for_disease_countries	Every 6 h	AviationStack/OpenSky	Flight routes for disease-affected areas
compute_flight_risk_assessments	Daily (07:00)	Internal (Disease × Aviation)	Cross-border risk scoring
fetch_google_trends	Daily (05:30)	Google Trends	Search interest indicators
fetch_flunet_data	Weekly (Tuesday 04:30)	WHO FluNet	Influenza surveillance data
backfill_embeddings	Daily (02:00)	Voyage AI	pgvector semantic embeddings
cleanup_old_records	Weekly (Sunday 03:00–04:00)	Database	Data retention management

#### Frontend

The frontend provides a multi-layer geospatial dashboard combining ArcGIS Maps SDK and Leaflet (with heatmap, marker clustering, and wind velocity overlays), disease event management with review workflows, epidemiological curve visualization, and a predictions module for forecasting, anomaly detection, and R_t estimation. Bilingual support (English and Chinese) is provided throughout.

### Data sources and ingestion

EpiGIS Pro integrates data sources with heterogeneous geographic scopes (Table 2). Disease event extraction is global (56 countries via news APIs); WHO FluNet influenza surveillance covers 9 countries selected for continuous reporting; aviation route modelling spans 633 international routes across 28 airports worldwide; and Google Trends signals are collected for 6 geographic regions. Two domains are deliberately Australia-focused as a pilot deployment: weather observations (8 cities) and traffic congestion indicators (8 cities, 45 road segments). These two domains currently serve as contextual layers for visualisation and proximity queries and are NOT used as covariates in the predictive models reported in this paper; their integration as exogenous regressors is identified as a Future Direction (see §Future Directions). Predictive analytics evaluation is correspondingly stratified: seasonality validation across 7 of the 9 FluNet countries, forecasting evaluation across 4 countries with sufficient data volume (Australia, United States, Japan, Germany), R_t estimation across 6 countries, and cross-border risk scoring globally.

**Table 2:** Geographic scope of integrated data sources and their role in current analyses

Data domain	Geographic scope	Records	Role in current analyses
Disease events (Claude AI)	56 countries (global)	353 articles → 326 events	Extraction evaluation; semantic search; risk scoring input
WHO FluNet influenza	9 countries	2,868 weekly records	Seasonality (7 countries); forecasting (4); R_t (6)
Aviation routes	Global (633 routes, 28 airports)	4,828 risk scores	Cross-border risk assessment
Google Trends	6 regions	5,580 weekly records	Auxiliary regressor in forecasting
Weather observations	Australia only (8 cities)	237,277 records	Contextual layer only — NOT in predictive models
Traffic congestion	Australia only (8 cities, 45 segments)	76,334 records	Contextual layer only — NOT in predictive models

### Disease event data

Disease events are collected from six source categories:

1. **NewsAPI.org**: Global news headlines filtered by disease-related keywords, fetched twice daily (06:00, 18:00 UTC).

2. **WHO Disease Outbreak News (DON)**: Official WHO outbreak reports via RSS feed, fetched twice daily (06:05, 18:05 UTC).

3. **Google News RSS**: Regional news feeds targeting disease-related content, fetched twice daily.

4. **GNews API**: Supplementary news aggregation service, fetched twice daily.

5. **Reddit**: Public posts from health-related subreddits with disease keyword filtering, fetched twice daily.

6. **China CDC**: Weekly infectious disease surveillance bulletins (Monday 04:00 UTC).

Each raw article is processed through the Claude AI extraction pipeline (described below) or stored in Amazon S3 for manual review when extraction confidence is low.

### WHO FluNet influenza surveillance data

WHO FluNet provides weekly influenza laboratory surveillance data from national influenza centres participating in GISRS [24]. Our dataset comprises 2,868 records across 9 countries (Australia, United States, Japan, Germany, France, Brazil, South Africa, United Kingdom, China) spanning ISO weeks from December 2019 to February 2026 (approximately 6.2 years per country). Fields include total influenza-positive specimens (inf_all), influenza A subtypes (A/H1N1pdm09, A/H3N2, A/H5), influenza B lineages (B/Yamagata, B/Victoria), and RSV detections.

### Google trends digital surveillance signals

Google Trends search interest data for 10 health-related keywords (e.g., “flu symptoms”, “dengue outbreak”) across 6 geographic regions are collected and stored as weekly time series (5,580 records). These serve as auxiliary regressors for forecasting models.

### Environmental data

Weather observations are collected from OpenWeatherMap One Call API 3.0 (primary) with Open-Meteo as a free fallback, covering 8 Australian cities at sub-hourly resolution. The dataset comprises 237,277 records including temperature, humidity, rainfall, wind speed/direction, atmospheric pressure, UV index, and cloud cover. Both current observations and 5-day forecast data are ingested.

### Aviation and mobility data

International flight route data are sourced from AviationStack API and OpenSky Network, covering 633 active routes across 28 monitored airports in Australia, Asia-Pacific, Middle East, Europe, and the Americas. Flight risk assessments (4,828 records) are computed daily by linking active disease events to departure countries and scaling by estimated daily passenger volumes.

Traffic congestion indicators are collected from HERE Traffic API v7, monitoring 45 urban road segments across 8 Australian cities at 15-minute intervals (76,334 records). Congestion levels are derived from the HERE jamFactor metric.

### AI-powered disease event extraction

The extraction pipeline uses Anthropic’s Claude AI (claude-3-haiku-20240307) with a structured prompt engineering approach. The prompt instructs the model to extract the following fields from each article:

• **Disease type**: Specific pathogen identification guided by a reference list of common infectious diseases, with the model capable of recognizing additional disease types beyond the reference list.

• **Location**: Geographic place name with latitude/longitude geocoding.

• **Case and death counts**: Explicit numerical values only (no estimation).

• **Severity**: Four-level classification (LOW/MEDIUM/HIGH/CRITICAL) based on case counts and official declarations.

### Standardised epidemiological metadata

- Priority level (E2): ROUTINE, ALERT, URGENT, or EMERGENCY
- Symptoms (E3): Structured symptom extraction (fever, cough, hemorrhage, etc.)
- Zoonotic indicator (E4): Animal-origin classification
- Syndrome (E5): ILI, SARI, ARI, GI, Hemorrhagic, Neurological, Rash, or Other

### Semantic search with vector embeddings

**Fig. 2:**
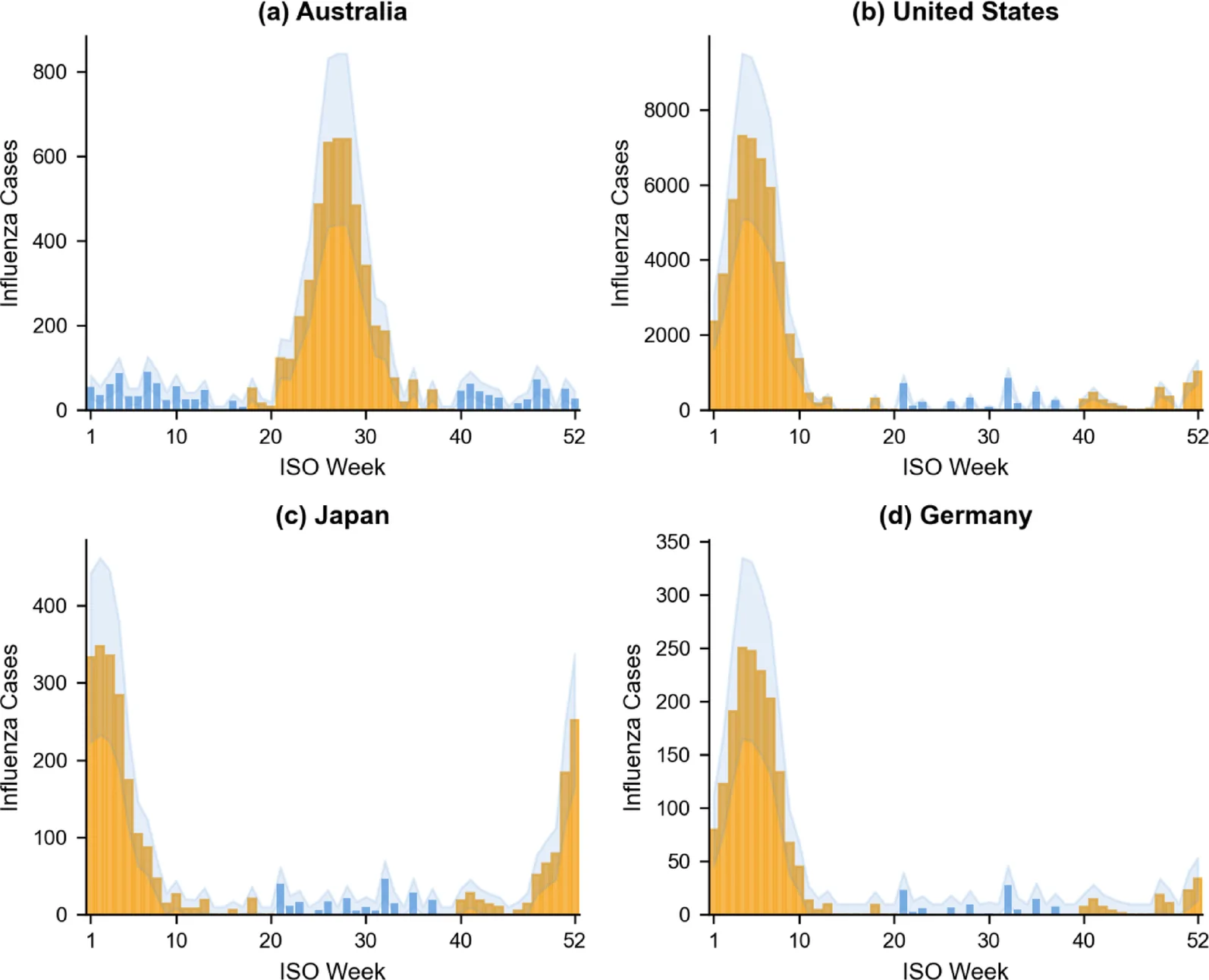
Seasonal influenza profiles for (**a**) Australia, (**b**) United States, (**c**) Japan, and (**d**) Germany, showing mean weekly case counts (bars) with standard deviation error bars across 6.2 years of WHO FluNet data. Southern Hemisphere countries peak during weeks 18–27 (austral winter), while Northern Hemisphere countries peak during weeks 50–5 (boreal winter)

Disease event descriptions are encoded into 512-dimensional dense vector embeddings using the Voyage AI voyage-3-lite embedding model [29], a transformer-based sentence embedding architecture in the lineage of sentence-transformer models (see footnote). These embeddings are stored in PostgreSQL using the pgvector extension with Hierarchical Navigable Small World (HNSW) indexes, enabling efficient approximate nearest-neighbor search for semantically similar disease events. This supports two key capabilities: (1) semantic search, where natural language queries retrieve relevant events beyond keyword matching (Fig. 2), and (2) similar event retrieval, where the system identifies historical events with comparable characteristics.

### Machine learning modules

#### Time-series forecasting

We employ the Prophet algorithm [25] for influenza case forecasting on FluNet data. Three model variants are compared:

##### Seasonal Naive (baseline):

Predicts future values as the same-week-last-year observation, serving as the reference for skill score computation.

$$\:{\widehat{y}}_{t+h}={y}_{t+h-52}$$

##### Prophet Multiplicative (proposed):

Uses raw (untransformed) case counts with multiplicative seasonality and custom Fourier terms.

Prophet(

yearly_seasonality=False,

seasonality_mode=’multiplicative’,

changepoint_prior_scale = 0.05,

seasonality_prior_scale = 15.0

)

model.add_seasonality(name=’yearly’, period = 365.25, fourier_order = 10)

##### Prophet Log-Additive (comparison)

Applies log1p transformation with additive seasonality, representing the conventional approach.

Evaluation uses rolling-origin cross-validation with an expanding training window (minimum 104 weeks), 5 folds, and forecast horizons of 4, 8, and 12 weeks. Metrics include root mean square error (RMSE), mean absolute error (MAE), mean absolute percentage error (MAPE), and skill score relative to the seasonal naive baseline:

$$\:\mathrm{Skill\:Score}=1-\frac{{\mathrm{RMSE}}_{\mathrm{model}}}{{\mathrm{RMSE}}_{\mathrm{baseline}}}$$

where positive values indicate improvement over the baseline.

### Anomaly detection

Anomaly detection employs a multi-method ensemble:

1. **Seasonal Z-score**: For each ISO week, a multi-year baseline (mean and standard deviation) is computed from historical FluNet data. Current values deviating beyond a configurable threshold (default z = 2.5) are flagged.

2. **Interquartile range (IQR)**: Log-transformed values outside 1.5× IQR are flagged.

3. **Isolation Forest**: An unsupervised ensemble method using features including log-transformed case counts, test positivity rate, week-of-year, and influenza A/B ratio. Default contamination is set to 5%.

A data point is classified as anomalous when 2 or more methods concur. Evaluation is performed against known outbreak peak periods documented by WHO and national CDCs.

### Effective reproduction number (R_t) estimation

The effective reproduction number is estimated from weekly FluNet data using a ratio-based approach adapted for weekly granularity. Given weekly case counts $\:{C}_{t}$, R_t is approximated as:

$$\:{R}_{t}=\frac{{C}_{t}}{\sum\:_{k=1}^{w}{C}_{t-k}\cdot\:{\omega\:}_{k}}$$

where $\:{\omega\:}_{k}$ is a discretized serial interval distribution (gamma-distributed with mean 3 days for influenza) and * w* is the smoothing window (default 4 weeks). R_t values are capped at 10.0 to prevent extreme ratios from low-count weeks.

### Seasonality analysis

Seasonal profiles are computed by aggregating FluNet data by ISO week across all available years, producing mean and standard deviation estimates for each of the 52 epidemiological weeks. Two complementary metrics are reported: *seasonal strength*, defined as the seasonal amplitude (maximum minus minimum weekly mean) divided by the overall standard deviation, quantifying the magnitude of the seasonal signal relative to background variability; and the *peak-to-trough ratio*, computed as the mean of the four highest weekly averages divided by the mean of the four lowest weekly averages, reflecting the fold-change between peak and off-season activity. Peak weeks are validated against known hemisphere-concordant influenza timing: weeks 40–52 and 1–20 (October–May) for the Northern Hemisphere and weeks 18–39 (May–September) for the Southern Hemisphere.

### Cross-border disease spread risk assessment

Flight risk assessments integrate disease event data with aviation route information. For each active flight route, a risk score (0–100) is computed as a function of:

- Active disease case counts in the departure country.
- Estimated daily passenger volume on the route.
- Disease severity classification.

**Fig. 3:**
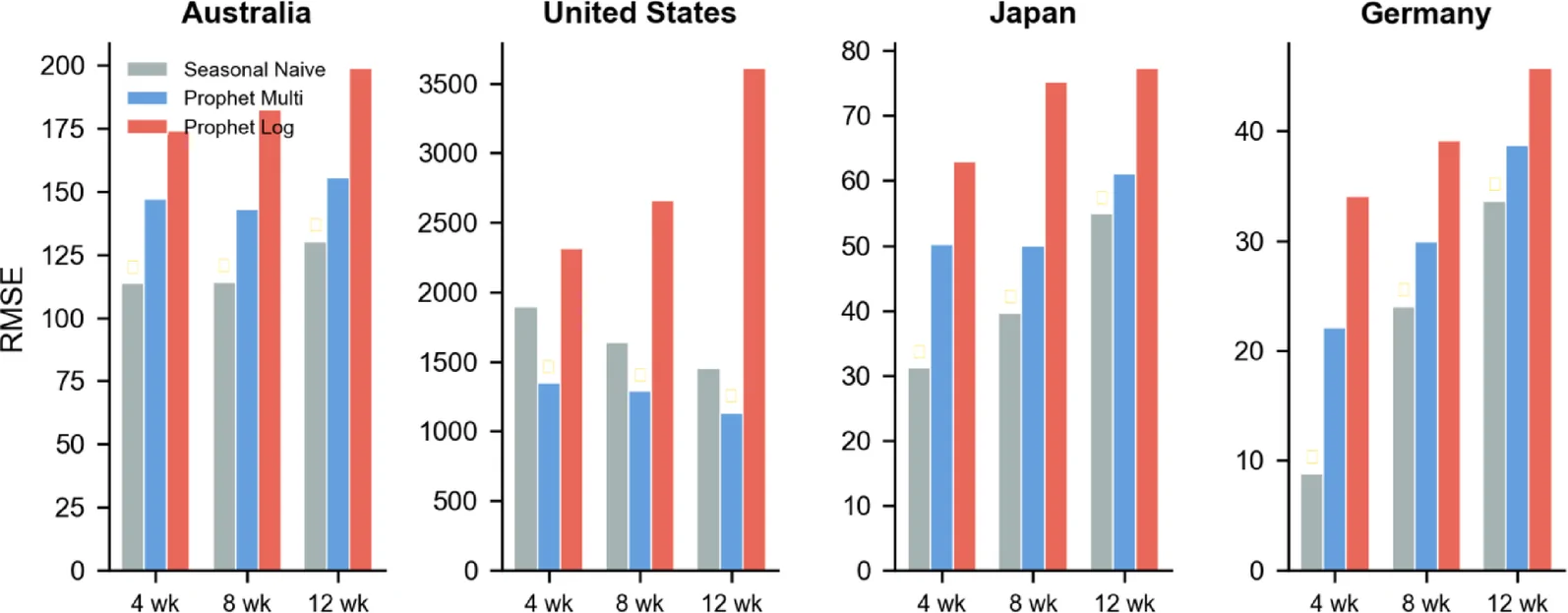
RMSE comparison across three forecasting models—Seasonal Naive (gray), Prophet Multiplicative (blue), and Prophet Log-Additive (red)—for four countries (Australia, United States, Japan, Germany) at 4-, 8-, and 12-week horizons. Prophet Multiplicative achieves the lowest RMSE for the United States across all horizons, while Seasonal Naive remains the best performer for the other three countries

Risk levels are categorized as LOW (0–19), MEDIUM (20–49), HIGH (50–74), and CRITICAL (≥ 75). For map visualization, a finer five-level color gradient is applied (green, yellow, orange, deep orange, red) at thresholds of 20, 40, 60, and 80 to enhance visual differentiation (Fig. 3). Assessments are recomputed daily.

### Spatial analysis

**Fig. 4:**
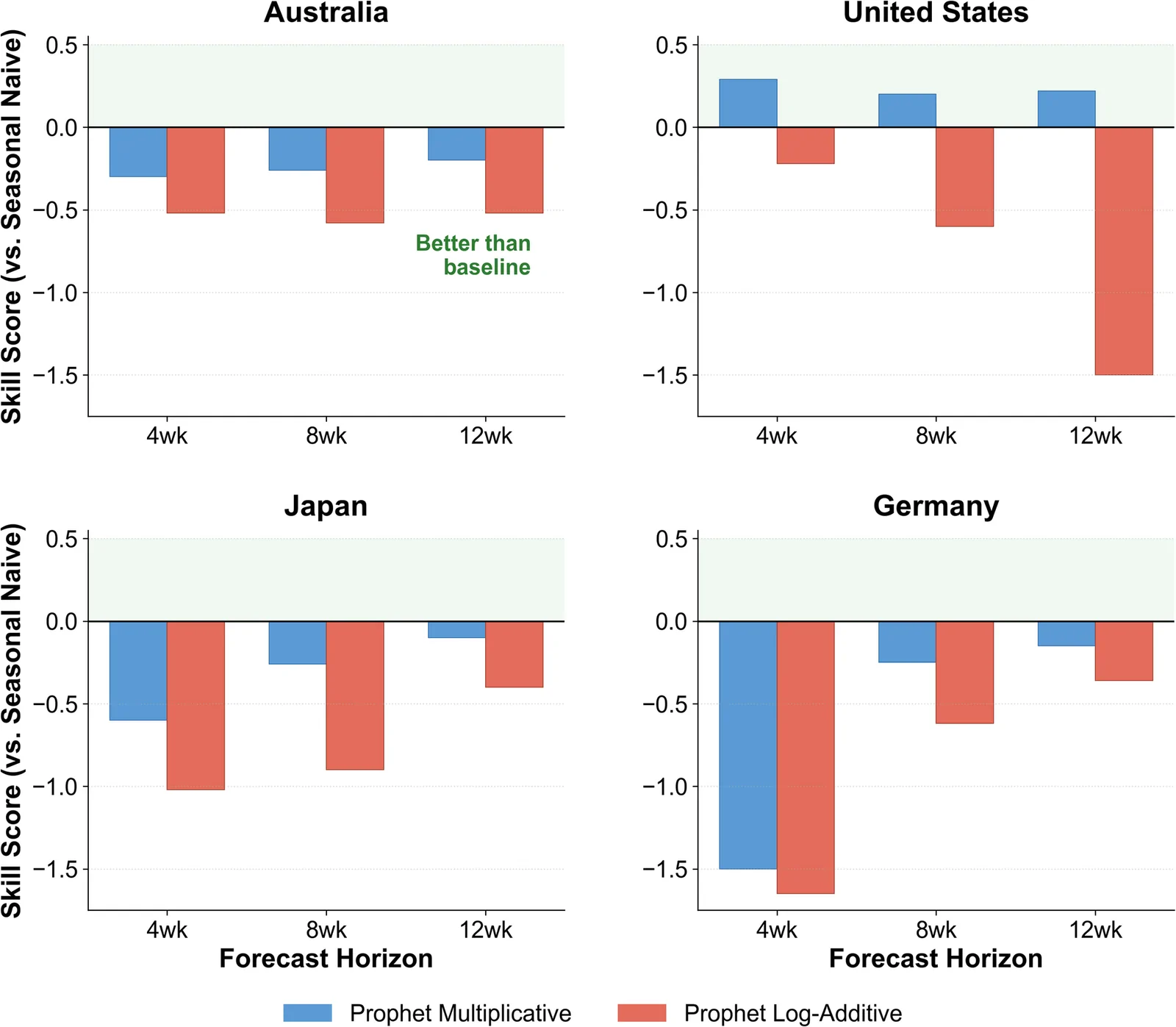
Forecast skill scores (relative to seasonal naive baseline) for Prophet Multiplicative (blue) and Prophet Log-Additive (red) across four countries at 4-, 8-, and 12-week horizons. Positive values (green-shaded region) indicate improvement over the baseline. Only the United States shows consistently positive skill scores for Prophet Multiplicative, while Log-Additive performs worse than baseline across all countries and horizons

The platform implements six PostGIS-enabled spatial analysis capabilities that operate directly on the geometry-typed disease event records and other location-aware tables (Fig. 4). All spatial functions execute server-side using PostGIS GiST indexes for efficient retrieval at interactive latencies.

#### Buffer-based proximity search

Given a query point and user-defined radius (default 50 km), the system retrieves all disease events whose geometry lies within that radius using the PostGIS function ST_DWithin (a distance-based predicate that returns true when two geometries are within a specified distance, leveraging spatial indexes). Distances are computed in metres on the geography type (WGS-84). Results are ranked by ST_Distance and returned with full event metadata (Fig. 4a).

#### Region containment

Events falling within an administrative polygon are retrieved via ST_Within and ST_Intersects predicates, supporting country-, state-, and city-level aggregation. Polygon boundaries are sourced from Natural Earth Data and stored as MultiPolygon geometries.

#### Density-based spatial clustering

The platform applies the DBSCAN (Density-Based Spatial Clustering of Applications with Noise) algorithm via PostGIS ST_ClusterDBSCAN, which assigns events to clusters based on two parameters: ε (the maximum neighbourhood distance, configurable; default 300 km in geographic CRS) and minPts (the minimum number of points required to form a dense region; default 3). Events that satisfy neither condition are labelled noise. For each detected cluster, the convex hull (ST_ConvexHull) and centroid (ST_Centroid) are computed for visualisation (Fig. 3c).

#### Per-country spatial summary

For each country with two or more events, the platform computes a convex hull outlining the spatial extent of disease events, a centroid label, and a circular spread radius (maximum point-to-centroid distance). Countries are colour-graded by event count using a five-level scheme (yellow < 5, orange 5–9, deep orange 10–19, red 20–49, dark red ≥ 50; Fig. 4b).

#### Healthcare facility proximity

Nearest-neighbour queries against a healthcare facility table return the k closest facilities (default k = 5) to a disease event location using the <-> KNN distance operator on PostGIS geometry indexes.

#### Weather context join

For Australian disease events (the geographic region with weather coverage), the nearest weather observation within a 50 km buffer is joined for contextual display alongside the event detail panel.

### Software dependencies and licensing

The platform’s primary visualisation stack uses ArcGIS Maps SDK for JavaScript 4.34 (developer use is free; production deployment is subject to Esri’s free-tier monthly map tile quota and may require a paid Esri account for high-volume deployments). To enable license-free deployment, equivalent map functionality—including base tiles, marker clustering, and heatmap overlays—is fully implemented through the Leaflet 1.9/react-leaflet 5.0 fallback layer (BSD-2-Clause licence) and is the default when no Esri credentials are configured. All other platform components are open-source under permissive licences: Django REST Framework (BSD-3), PostgreSQL/PostGIS (PostgreSQL Licence/GPL-2), pgvector (PostgreSQL Licence), Celery (BSD-3), Prophet (MIT), React (MIT), and Docker Engine (Apache-2.0). The Anthropic Claude and Voyage AI APIs require commercial accounts for the disease event extraction and semantic search modules; both modules degrade gracefully when API credentials are absent (a keyword-based fallback extractor replaces Claude, and lexical full-text search replaces vector similarity).

### Evaluation framework

A systematic evaluation framework was implemented as a set of API endpoints enabling reproducible assessment across six dimensions:

1. **Data coverage**: Record counts, temporal spans, and geographic coverage across all data sources
2. **AI extraction quality**: Processing success rate and field completeness across 10 extraction fields.
3. **Seasonality validation**: Hemisphere-concordant peak timing accuracy across 7 countries.
4. **Forecast accuracy**: Multi-model rolling-origin cross-validation across 4 countries at 3 horizons.
5. **Anomaly detection performance**: Precision, recall, and F1-score against documented outbreak periods.
6. **R_t estimation consistency**: Onset-period vs. trough-period R_t comparison for epidemiological plausibility.

## Results

### Data integration coverage

**Fig. 5:**
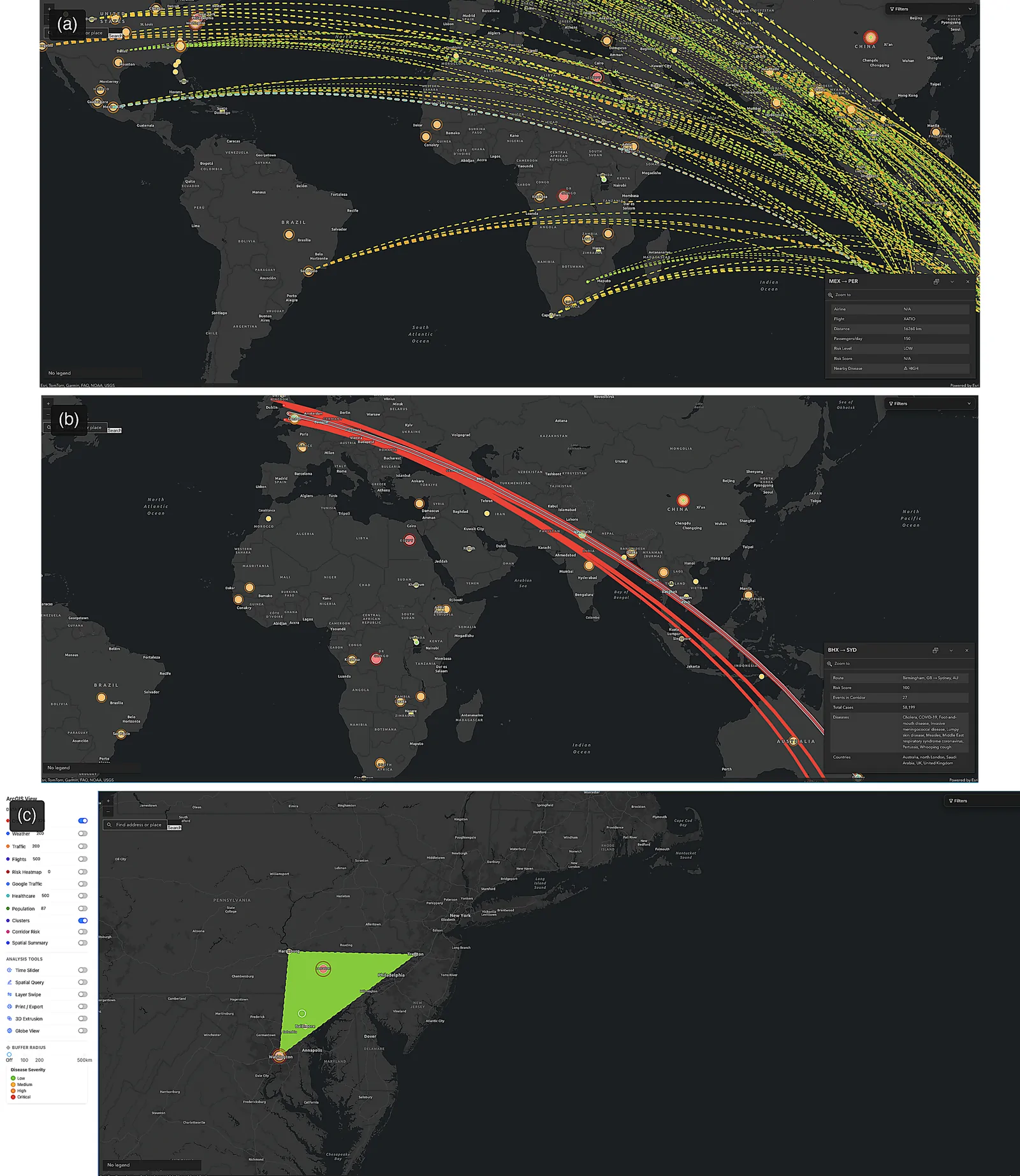
ArcGIS geospatial visualization capabilities. (**a**) Global flight route network with disease event markers: route colors follow a five-level risk gradient—green (risk score < 20, low), yellow (20–39), orange (40–59), deep orange (60–79), and red (≥ 80, high)—derived from PostGIS spatial analysis of disease events within each flight corridor buffer zone. Markers show AI-extracted disease events color-coded by severity. (**b**) Corridor risk detail view showing high-risk flight routes (red) originating from Europe to the Asia-Pacific region, with the information panel displaying route-specific risk scores, linked disease events within the corridor buffer, and affected countries. (**c**) Spatial clustering analysis (ST_ClusterDBSCAN) of disease events in the US Northeast, showing the convex hull boundary (green shaded polygon) of a detected cluster; each cluster is assigned a distinct color for visual differentiation. The left panel displays 11 toggleable map layers and spatial analysis tools including time slider, spatial query, and 3D extrusion

Table 3; Fig. 3 summarize the data integrated into the EpiGIS Pro platform as of February 2026. The platform dashboard (Fig. 5a) provides a real-time overview of all integrated data domains through four KPI summary cards and interactive trend visualizations.

**Fig. 6:**
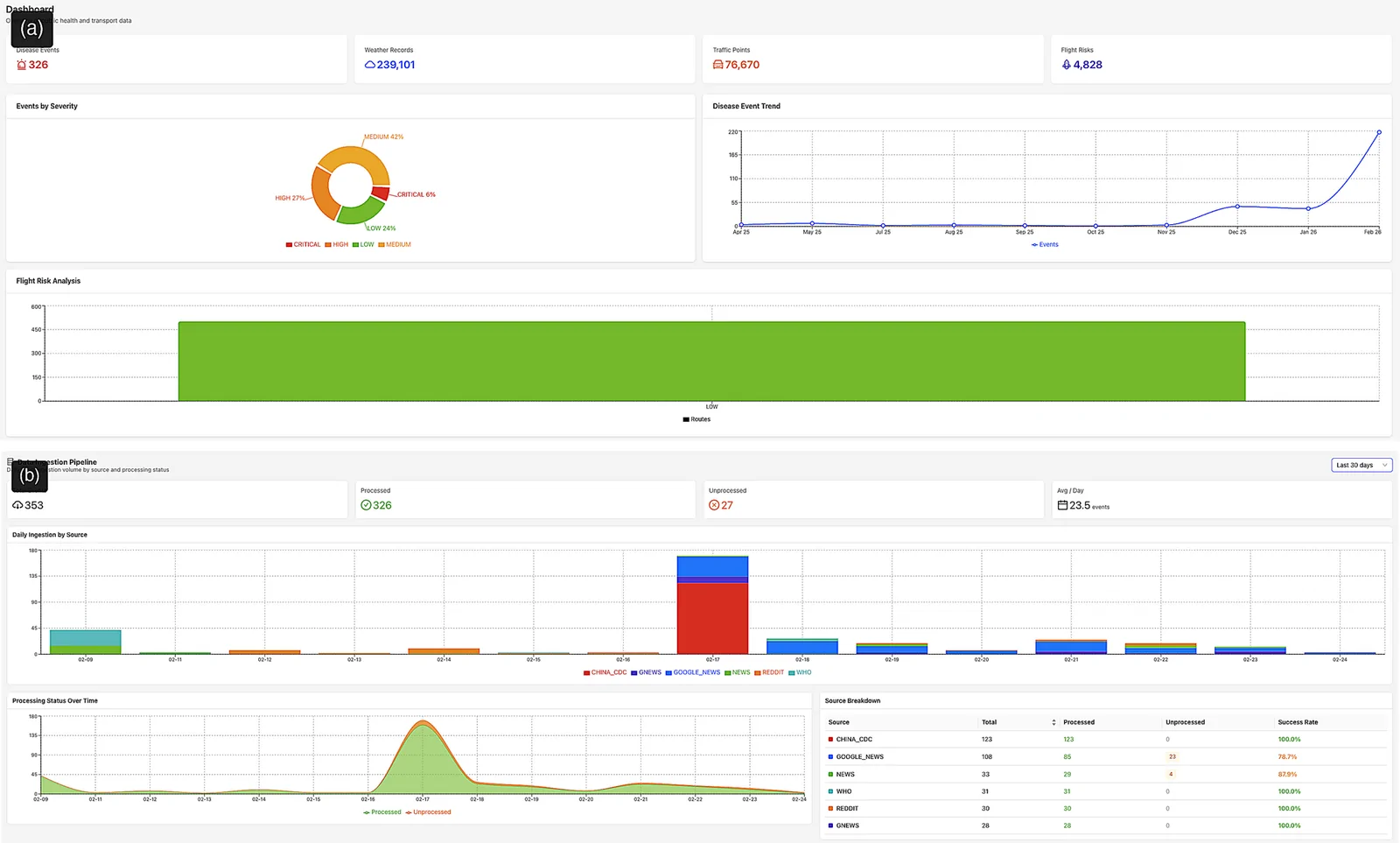
Platform dashboard views. (**a**) Dashboard overview displaying four KPI summary cards (Disease Events, Weather Records, Traffic Points, Flight Risks), a severity distribution donut chart, a disease event trend line showing temporal distribution of extracted events from April 2025 to February 2026, and a flight risk analysis bar chart. Dashboard values reflect real-time data and may differ slightly from the snapshot reported in Table 3. (**b**) Data ingestion pipeline monitoring panel showing total events processed (353), processing success rate (92.4%), average extraction time (23.5 s), daily ingestion volume by source (stacked bar chart across six source categories), processing status over time, and a source breakdown table with per-source success rates

**Table 3:** Data source coverage summary

Data source	Records	Geographic coverage	Temporal range	Usage
Disease Events (AI-extracted)	353	56 countries, 104 disease types	Apr 2025 – Feb 2026	Contextual + Risk scoring
WHO FluNet	2,868	9 countries	Dec 2019 – Feb 2026	Model (forecasting, anomaly detection, R_t)
Google Trends	5,580	6 regions, 10 keywords	Nov 2025 – Feb 2026	Model (auxiliary regressor)
Weather Observations	237,277	8 Australian cities	Multi-source	Contextual
Aviation Routes	633	28 airports globally	Active routes	Risk scoring
Flight Risk Assessments	4,828	28 airports globally	Daily computed	Risk scoring
Traffic Indicators	76,334	45 segments, 8 cities	15-min intervals	Contextual
**Total**	**327**,**873**			

Usage indicates whether data are used in predictive models (Model), for visualization and spatial queries (Contextual), or for risk scoring (Risk scoring)

The bold formatting in Table 3 ("Data source coverage summary") is applied to the summary 'Total' row at the bottom of the table (Total / 327,873),where it indicates the aggregate sum of the Records column across all seven data sources (disease events, WHO FluNet, Google Trends, weatherobservations, aviation routes, flight risk assessments, and traffic indicators). This follows standard tabular convention for visually distinguishingsummary/aggregate rows from individual data rows; bold is not used to denote statistical significance, best-performing values, or other analyticalemphasis.

### AI-powered disease event extraction

**Fig. 7:**
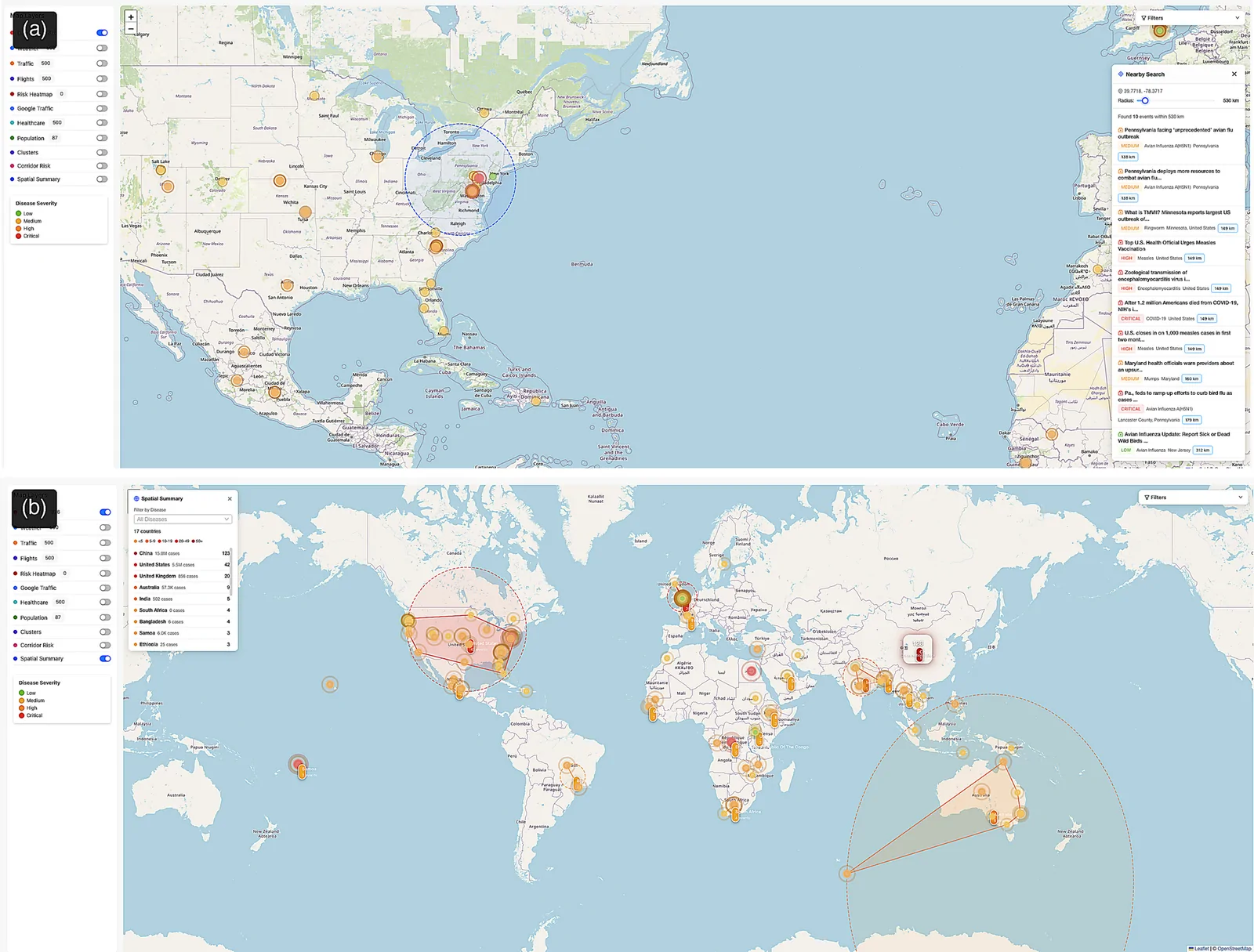
PostGIS spatial analysis capabilities in map view. (**a**) Spatial query with proximity search: a circular buffer zone (blue) centered on a selected location identifies nearby disease events within a user-defined radius, with the right-hand panel listing matched events ranked by distance and displaying event metadata including disease type, severity, and case counts. (**b**) Spatial summary layer showing per-country disease spread: each country is represented by a labeled centroid marker (country name and event count), a convex hull polygon outlining the spatial extent of disease events, and a dashed spread-radius circle. Colors follow a five-level event-count gradient—yellow (< 5 events), orange (5–9), deep orange (10–19), red (20–49), and dark red (≥ 50)—enabling rapid identification of high-burden regions

**Fig. 8:**
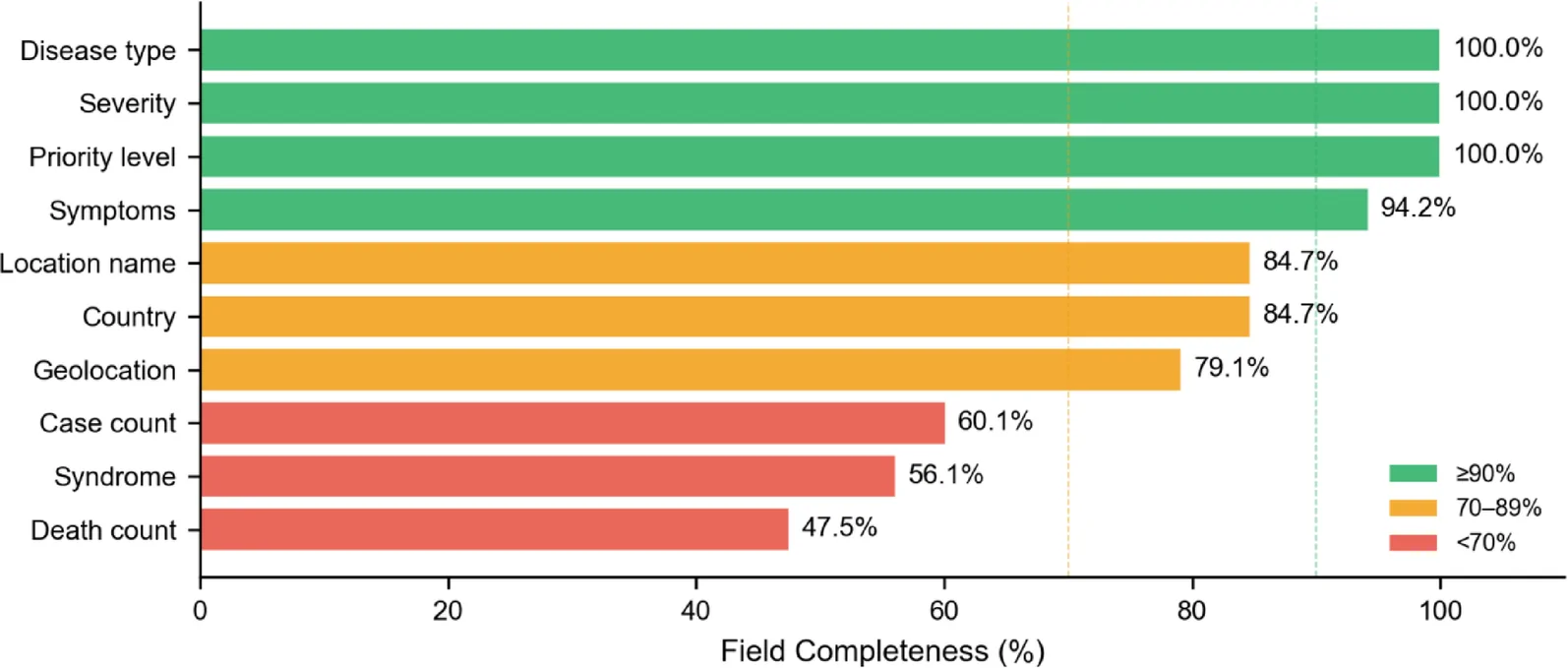
AI extraction field completeness for 326 successfully processed disease events. Horizontal bars show the percentage of events for which each metadata field was populated by Claude AI. Disease type, severity, and priority classification achieve 100% completeness, while quantitative fields (case count 60.1%, death count 47.5%) exhibit lower completeness due to incomplete reporting in source articles

Of 353 ingested articles, 326 (92.4%) were successfully processed by Claude AI with an average extraction time of 23.5 s per article. The data ingestion pipeline monitoring panel (Fig. 6b) provides per-source processing statistics and success rates. Figure 7 shows the disease event management interface where all AI-extracted events can be filtered, reviewed, and validated. Table 4; Fig. 8 present field completeness rates.

**Table 4:** AI extraction field completeness for processed disease events (*n* = 326)

Field	Completeness (%)	Description
Disease type	100.0	Specific pathogen identification
Severity	100.0	LOW/MEDIUM/HIGH/CRITICAL classification
Priority level	100.0	Priority classification
Symptoms	94.2	Structured symptom list
Location name	84.7	Geographic place name
Country	84.7	Country identification
Geolocation	79.1	Latitude/longitude coordinates
Case count	60.1	Explicit numerical case count
Syndrome	56.1	Syndrome classification
Death count	47.5	Explicit numerical death count

Among the 326 successfully processed events, the system identified 104 consolidated disease types, with measles (*n* = 79, 24.2%), COVID-19 (*n* = 15, 4.6%), and avian influenza including A(H5N1) (*n* = 12, 3.7%) being the most frequently detected (Table S1). Events spanned 56 countries, with the highest volumes from China (*n* = 113, 34.7%), United States (*n* = 46, 14.1%), and United Kingdom (*n* = 19, 5.8%). Severity distribution was MEDIUM (42.3%), HIGH (27.3%), LOW (24.2%), and CRITICAL (6.2%).

### Influenza seasonality validation

**Fig. 9:**
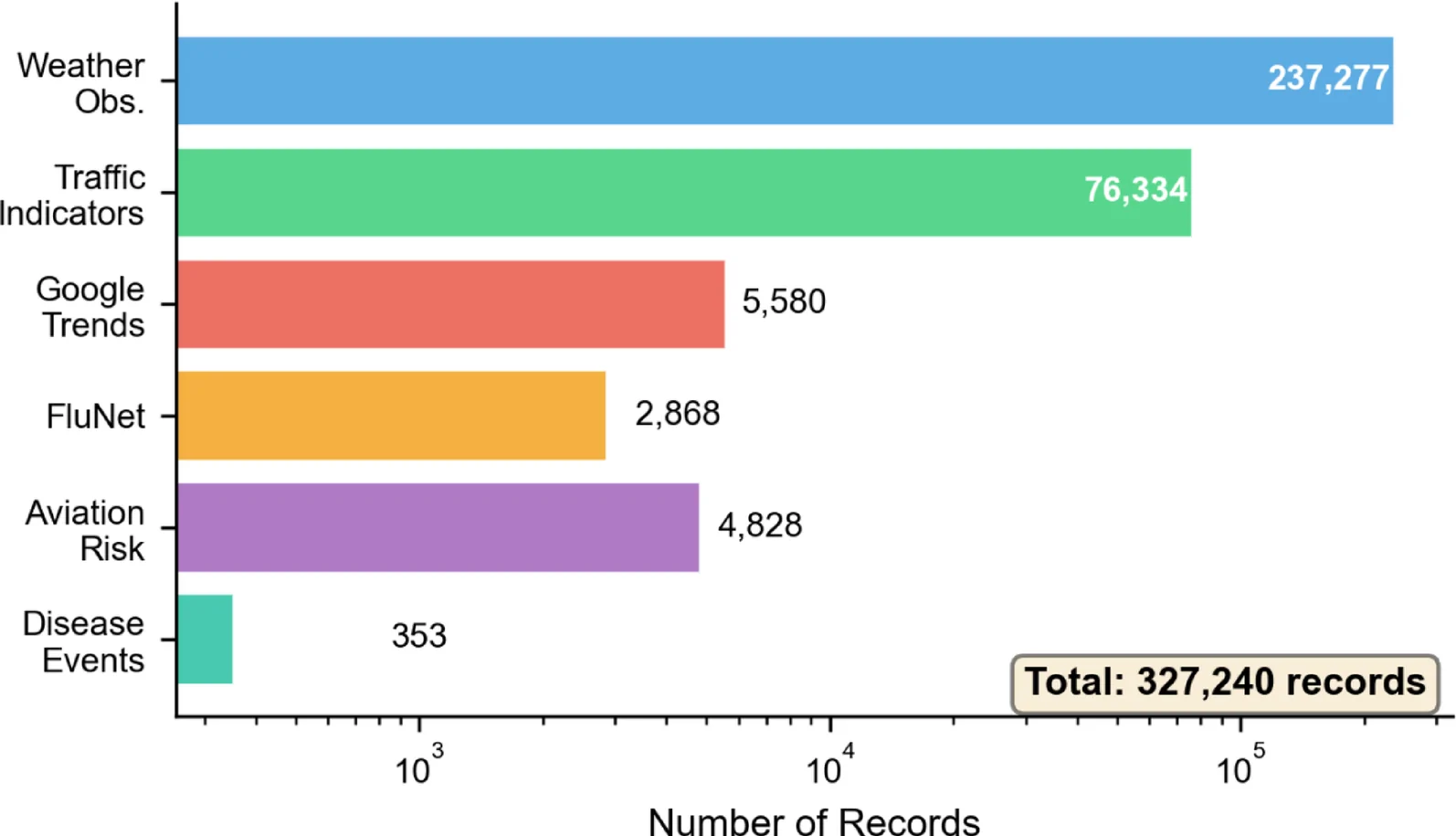
Data source record counts across the six integrated data domains, illustrating the scale and heterogeneity of the EpiGIS Pro data warehouse. Weather observations constitute the largest domain, followed by traffic indicators and Google Trends. Record counts reflect real-time data at the time of capture and may differ slightly from the snapshot reported in Table 3

Seasonality analysis was performed on WHO FluNet data for 7 countries spanning both hemispheres (Table 5; Fig. 9). All countries exhibited hemisphere-concordant influenza peak timing (100% accuracy).

**Table 5:** Influenza seasonality analysis across 7 countries using WHO FluNet weekly data

Country	Hemisphere	Data (years)	Peak Weeks	Seasonal strength	Peak-trough ratio‡	Match
Australia	Southern	6.2	26–27	2.38	17.0	✓
Brazil	Southern	6.2	18–21	1.55	6.4	✓
South Africa	Southern	6.2	21–22	2.49	70.5	✓
United States	Northern	6.2	1–5	1.79	48.7	✓
Japan	Northern	6.2	50–2	1.83	27.1	✓
Germany	Northern	6.2	4–5	2.18	457.3	✓
France	Northern	6.0	4–5	2.36	—†	✓

Southern Hemisphere countries (Australia, Brazil, South Africa) showed peak influenza activity during weeks 18–27 (May–July), consistent with the austral winter. Northern Hemisphere countries (United States, Japan, Germany, France) peaked during weeks 50–5 (December–January), consistent with the boreal winter. Germany exhibited the highest peak-to-trough ratio (457.3), reflecting near-zero inter-season baseline activity. France’s peak-to-trough ratio could not be computed (†) due to zero-value trough weeks. Peak-to-trough ratios (‡) are computed as the mean of the four highest weekly averages divided by the mean of the four lowest weekly averages, and may therefore differ slightly from single-week peak/trough values reported in Table S3. The United Kingdom and China were excluded from seasonality validation due to incomplete reporting periods and data gaps that prevented reliable seasonal profile computation.

### Time-series forecast performance

**Fig. 10:**
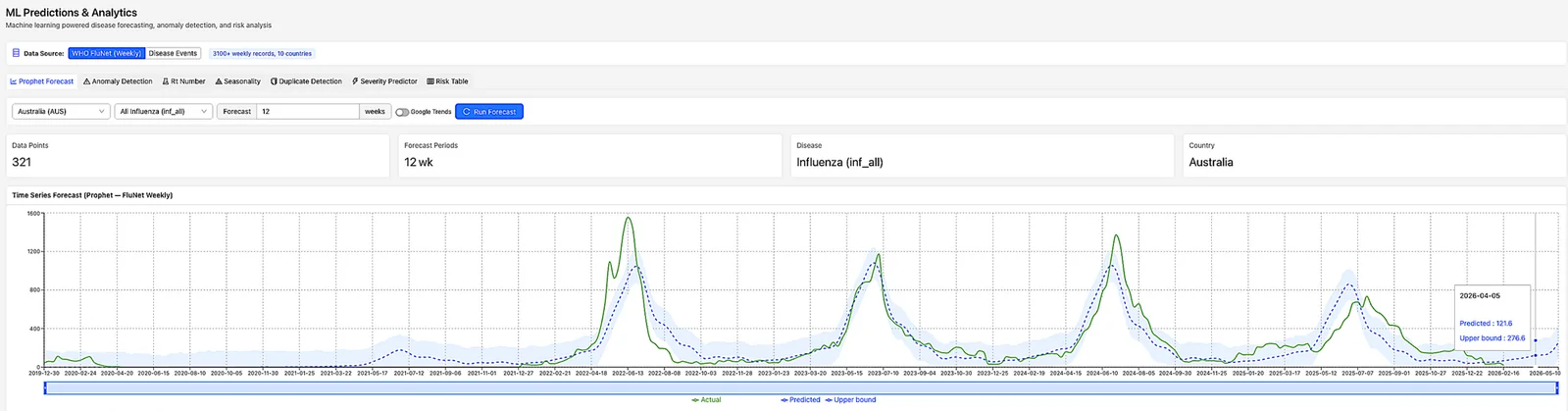
Prophet time-series forecast interface for Australia influenza (inf_all). The upper panel shows parameter controls: country selector, disease type, forecast horizon (12 weeks), and frequency (weekly). The time-series chart displays historical observed values (blue solid line), Prophet multiplicative model predictions (green dashed line), and 95% confidence interval (shaded band) from 2019 to 2026. Seasonal influenza peaks are clearly visible, with the model capturing both amplitude and timing of austral winter surges. The latest forecast point (2026-04-06) shows predicted value 1,318 with upper bound 2,114

**Fig. 11:**
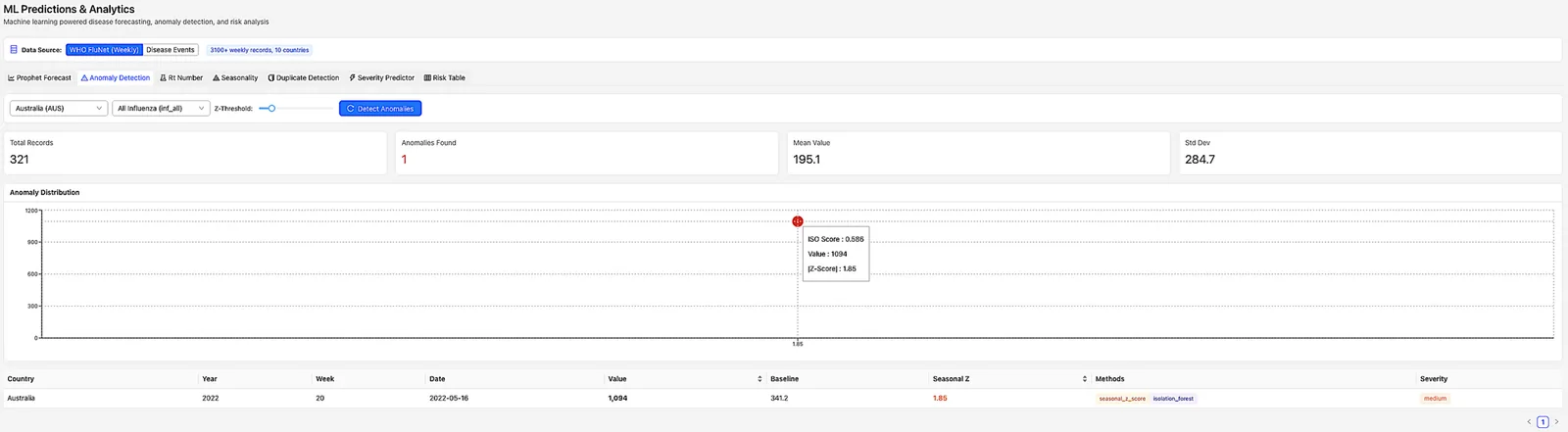
Anomaly detection interface for Australia influenza data. Four KPI cards display Total Records (321), Anomalies Found (1), Mean Value (195.1), and Standard Deviation (284.7). The anomaly distribution chart highlights the detected anomaly (red dot) with a tooltip showing ISO Score 0.586, Value 1,094, and |Z-Score| 1.85. The table below lists the anomaly detail: Australia, 2022 Week 20, with seasonal Z-score and isolation forest methods both flagging the observation, classified as medium severity

Multi-model cross-validation was performed across 4 countries (Australia, United States, Japan, Germany) with 5-fold rolling-origin evaluation at 4-, 8-, and 12-week horizons (Table 6). These countries were selected based on having the most complete and consistently reported FluNet data; the remaining five countries (Brazil, South Africa, France, United Kingdom, China) were excluded from forecasting due to shorter continuous reporting periods, intermittent data gaps, or insufficient data volume for reliable cross-validation. Figure 10 compares RMSE across the three models for each country and horizon. Figure 11 shows the Prophet forecast interface for Australia, demonstrating the model’s ability to capture seasonal amplitude and timing.

**Table 6:** Forecast performance comparison (RMSE) across three models and four countries

Country	Horizon	Seasonal Naive	Prophet Multi (SS)	Prophet Log (SS)
**Australia**	4 wk	**113.5**	147.2 (−0.30)	174.0 (−0.53)
	8 wk	**114.1**	143.0 (−0.25)	261.1 (−1.29)
	12 wk	**130.1**	164.9 (−0.27)	324.9 (−1.50)
**United States**	4 wk	1891.7	**1344.1** (0.29)	2314.9 (−0.22)
	8 wk	1637.9	**1286.8** (0.21)	2657.9 (−0.62)
	12 wk	1452.0	**1131.9** (0.22)	3608.2 (−1.49)
**Japan**	4 wk	**31.2**	50.2 (−0.61)	62.9 (−1.02)
	8 wk	**39.6**	49.9 (−0.26)	75.2 (−0.90)
	12 wk	**54.9**	61.1 (−0.11)	77.3 (−0.41)
**Germany**	4 wk	**8.8**	22.1 (−1.51)	34.0 (−2.86)
	8 wk	**24.0**	29.9 (−0.25)	39.1 (−0.63)
	12 wk	**33.6**	38.7 (−0.15)	45.7 (−0.36)

Bold indicates best performance per country-horizon. Skill scores (SS) are relative to seasonal naive baseline

**Fig. 12:**
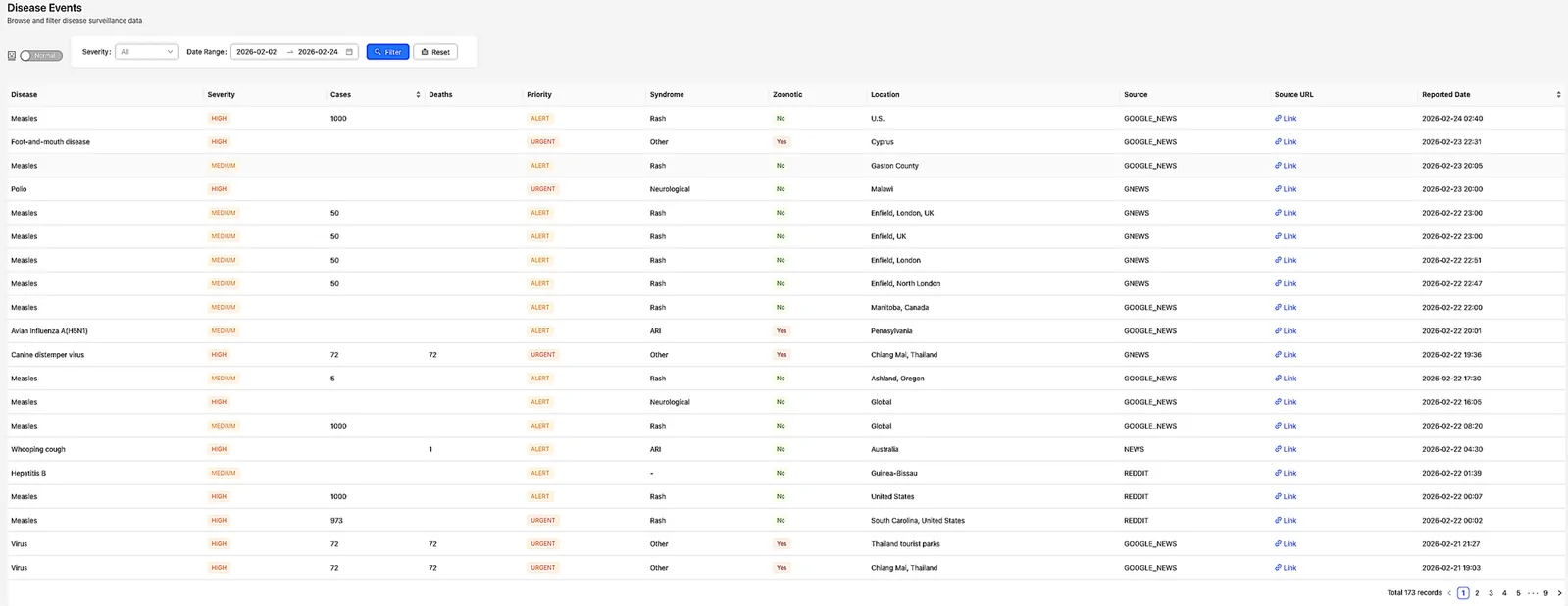
Disease event management interface showing the AI-extracted event registry. The filterable table displays 353 events with columns for disease type, severity (color-coded tags: Critical, High, Medium, Low), priority, cases, deaths, syndrome, zoonotic status, location, data source (GOOGLE_NEWS, GNEWS, NEWS, REDDIT), source URL, and reported date. Filters include severity level, date range, and view toggle (table/card). The interface supports manual review workflows for quality assurance of AI-extracted events

Prophet Multiplicative achieved positive skill scores only for the United States (0.21–0.29), where sufficient data volume and variability allowed the model to learn meaningful seasonal patterns (Fig. 12). For Australia, Japan, and Germany, the seasonal naive baseline outperformed both Prophet variants, though Prophet Multiplicative consistently outperformed Prophet Log-Additive. The log-transform approach substantially degraded forecast accuracy, with MAPE increasing by approximately 110–290% compared to the multiplicative approach across all countries and horizons (e.g., United States 4-week MAPE: 72.9% multiplicative vs. 159.1% log-additive). Complete forecast metrics including MAE, MAPE, symmetric mean absolute percentage error (sMAPE), and the coefficient of determination (R²) for all country-horizon combinations are provided in Table S2.

### Anomaly detection

**Fig. 13:**
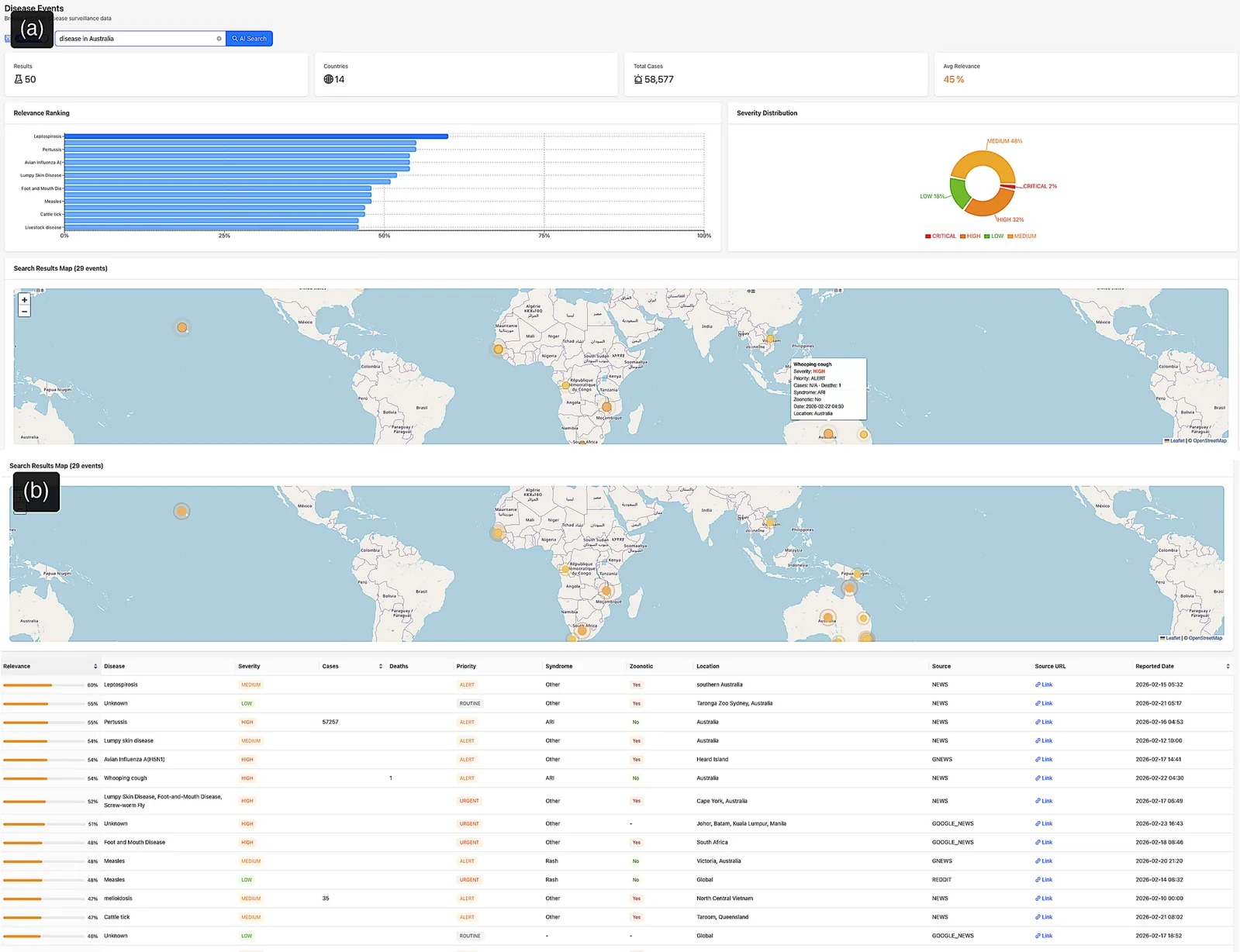
Semantic search powered by Voyage AI embeddings and pgvector. (**a**) Search results overview for the query “disease in Australia”: four KPI cards (Results 50, Diseases 14, Total Cases 58,577, Avg Relevance 45%), a horizontal bar chart ranking matched disease types by relevance score, a severity distribution donut chart, and a geographic map plotting the 29 matched events with popup metadata. (**b**) Detailed results view showing the ranked event list with relevance percentage bars, disease name, severity tags, cases, deaths, syndrome, zoonotic flag, location, source, and reported date. Each result includes a direct link to the source article

Anomaly detection was evaluated against known influenza outbreak peak periods in Australia (2022–2024). Table 7 shows performance at varying Z-score thresholds. The anomaly detection interface (Fig. 13) displays detected anomalies with both seasonal Z-score and isolation forest methods.

**Table 7:** Anomaly detection performance for Australia (WHO FluNet, inf_all metric)

Z-threshold	Detected	True Positives	Precision	Recall	F1
1.5	4	2	0.50	0.071	0.125
2.0	3	1	0.33	0.036	0.065
2.5	2	1	0.50	0.036	0.067

The multi-method ensemble achieved conservative anomaly detection, with high precision but low recall at the individual-week level. This reflects the challenge that “peak weeks” in known outbreaks often remain within 2 standard deviations of the seasonal baseline, as influenza exhibits high inter-annual variability. When evaluated at the outbreak-event level—i.e., whether at least one week within each known outbreak season was flagged—the ensemble detected 2 of 3 documented outbreak seasons (event-level recall: 67%), indicating that the system can identify the occurrence of anomalous seasons even when it does not flag every individual peak week. The anomaly detector is designed for early detection of unusual deviations rather than retrospective outbreak classification.

### R_t estimation

**Fig. 14:**
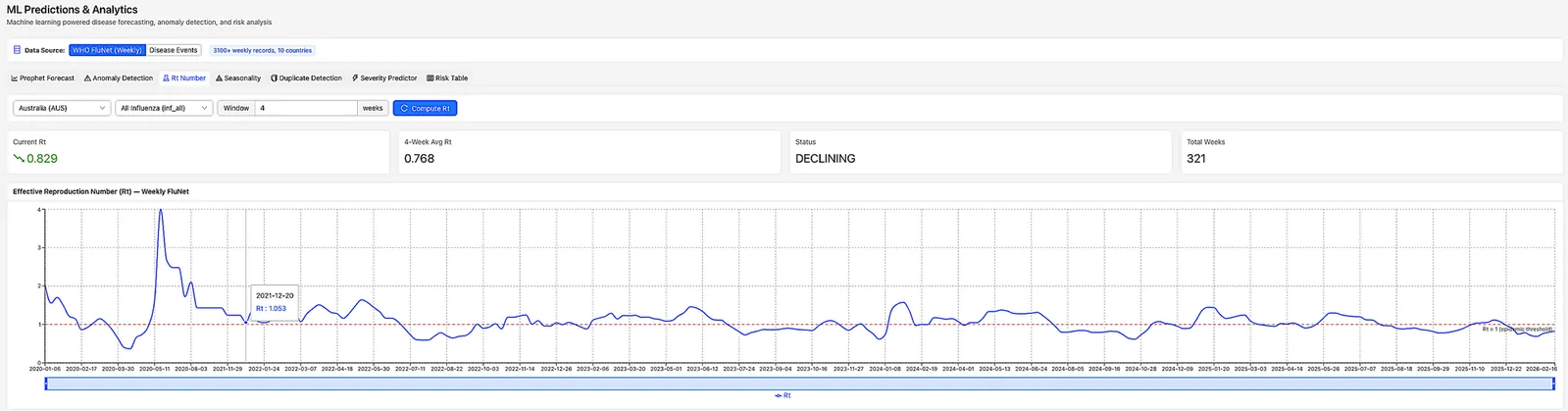
Effective reproduction number (R_t) estimation interface for Australia influenza. The upper panel shows the current R_t value (0.83), mean R_t (1.09), trend status (DECLINING), and total data points (321). The time-series chart displays the weekly R_t estimate from 2019 to 2026, with R_t briefly exceeding 1.0 during the 2021 COVID-influenced period (R_t = 1.063 on 2021-12-03) and subsequent stabilization below the epidemic threshold (R_t = 1.0)

Effective reproduction number estimation was performed for 6 countries with sufficient continuous weekly data (Table 8; Fig. 14); South Africa, the United Kingdom, and China were excluded due to sparse reporting weeks that produced unreliable R_t estimates. The R_t estimation interface provides real-time trend assessment with current R_t value, mean, and trend status.

**Table 8:** Effective reproduction number (R_t) estimation summary from WHO FluNet data

Country	Valid R_t Weeks	Current R_t	Status	Mean R_t	% Above 1.0	Onset > Trough R_t
Australia	249	0.83	Declining	1.09	58.6%	✓ (1.27 vs. 1.09)
Japan	219	0.81	Declining	1.49	53.9%	✓ (1.80 vs. 1.08)
Brazil	212	1.78	Growing	1.30	62.7%	—
Germany	225	1.02	Stable	2.26	68.4%	—
France	167	0.88	Declining	1.68	60.5%	—
United States	319	0.75	Declining	1.40	55.2%	✗ (1.10 vs. 1.47)

R_t estimation demonstrated epidemiologically plausible patterns for the majority of countries. Australia and Japan showed the expected pattern of higher R_t during epidemic onset compared to seasonal trough periods. The United States showed an inverted pattern (onset R_t 1.10 vs. trough R_t 1.47), likely attributable to the sustained high baseline transmission and the influence of atypical COVID-19-era seasons on the trough-period calculation. As of February 2026, Australia (R_t = 0.83), Japan (R_t = 0.81), and the United States (R_t = 0.75) show declining influenza transmission, while Brazil (R_t = 1.78) reflects its position in the Southern Hemisphere pre-peak seasonal buildup. Onset-versus-trough comparison was not computed for Brazil, Germany, and France due to insufficient separation between onset and trough periods in their available data.

## Discussion

### Principal findings

EpiGIS Pro demonstrates the feasibility of building an integrated disease surveillance platform that combines AI-driven information extraction, multi-source data fusion, and predictive analytics within a unified geospatial framework. Three key findings merit discussion.

First, the use of a large language model (Claude AI) for disease event extraction achieved 92.4% processing success with high field completeness for core surveillance fields (disease type: 100%, severity: 100%, symptoms: 94.2%). This compares favorably with traditional NLP approaches for disease extraction, where F1 scores typically range from 70 to 85% for named entity recognition tasks on biomedical text [9, 10]. The LLM approach eliminates the need for task-specific training data and supports extraction from multilingual sources, including Chinese-language CDC bulletins, without model retraining.

Second, the multi-model forecasting comparison revealed that no single model dominates across all geographic contexts. Prophet Multiplicative achieved meaningful skill improvement for the United States (skill score 0.21–0.29 vs. seasonal naive) but not for smaller-volume countries such as Germany and Japan. This finding aligns with the broader forecasting literature suggesting that model performance is data-volume-dependent and that simpler baselines often outperform complex models when training data are limited [26, 27]. Importantly, the log-transform approach widely used in epidemiological forecasting degraded accuracy substantially (approximately 110–290% MAPE increase across all countries), reinforcing the value of model selection and transformation analysis.

Third, the platform’s ability to link disease surveillance data with aviation routes for cross-border risk assessment represents a practical implementation of the conceptual frameworks proposed in pandemic preparedness literature [13, 14]. The integration of 633 flight routes with automated risk scoring—computed by linking departure-country disease activity with passenger volumes—provides actionable intelligence for public health decision-making (Fig. 3).

### Comparison with existing platforms

Compared with established surveillance systems, EpiGIS Pro offers several differentiating capabilities:

- **HealthMap** [7]: Provides automated aggregation but uses classical NLP; EpiGIS Pro’s LLM extraction achieves 100% completeness for core fields (disease type, severity, priority), whereas rule-based and classical NLP approaches typically yield variable completeness depending on source format [9, 10].
- **EIOS (WHO)** [8]: Focuses on signal detection; EpiGIS Pro adds predictive analytics, co-located environmental and mobility data layers, and aviation-based risk assessment.
- **FluSight (CDC)** [28]: Provides US-focused influenza forecasting; EpiGIS Pro covers 9 countries with multi-domain data integration.

### Strengths and Limitations

#### Strengths

First, EpiGIS Pro is among the first platforms to consolidate six heterogeneous data domains—disease events, influenza surveillance, environmental monitoring, aviation mobility, traffic congestion, and digital search trends—within a single analytical framework, enabling cross-domain situational awareness that existing platforms address only in isolation. Second, the LLM-based extraction pipeline achieved 92.4% processing success and 100% completeness for core epidemiological fields (disease type, severity, priority), demonstrating that general-purpose large language models can serve as effective structured extractors for disease surveillance without domain-specific fine-tuning. Third, the semantic search capability powered by Voyage AI embeddings and pgvector enables similarity-based retrieval across disease events, surpassing traditional keyword-based approaches used by existing surveillance systems. Fourth, the integrated predictive analytics suite—combining Prophet forecasting, seasonal Z-score anomaly detection, R_t estimation, and seasonality profiling—provides a comprehensive analytical toolkit accessible through a unified web interface and RESTful API. Fifth, the platform’s modular architecture allows independent scaling and extension of each data domain, and the evaluation framework—implemented as reproducible API endpoints—enables transparent performance assessment and model comparison. Sixth, the use of PostGIS spatial extensions supports proximity analysis, buffer queries, and spatial indexing, providing genuine GIS analytical capabilities beyond simple map visualization. Finally, Docker containerization and AWS ECS deployment ensure reproducibility across environments.

#### Limitations

Several limitations should be noted. First, the disease event dataset (353 events over 11 months) is small compared to established surveillance databases. Performance will improve as the automated ingestion pipeline accumulates data over time. Second, forecast performance varies substantially by country, with the seasonal naive baseline remaining competitive or superior for low-volume countries—a known challenge in epidemiological forecasting [27]. Third, the anomaly detection module showed low recall against known outbreak periods, reflecting the conservative multi-method ensemble and the challenge of distinguishing “outbreaks” from normal seasonal variation in countries with high inter-annual variability. Fourth, the cross-border risk assessment currently uses simple multiplicative scoring rather than mechanistic epidemiological transmission models such as the susceptible–exposed–infectious–recovered (SEIR) compartmental framework [30], which is planned for future development. Fifth, the weather and traffic data are deliberately scoped to an Australian pilot (8 cities for both domains) and serve solely as contextual layers for visualisation and spatial proximity queries; they are not used as covariates in the predictive models reported here, which rely exclusively on FluNet case counts and Google Trends regressors. For all non-Australian countries, the predictive analytics (forecasting, anomaly detection, R_t estimation) operate on FluNet and disease event data alone, and platform users in those regions are presented with case-volume and aviation-risk visualisations without the weather and traffic contextual overlays. This scope limitation is restated in Methods (see Table 2a) to avoid the appearance of a global environmental modelling claim.

### Future directions

Planned extensions include: (1) geographic expansion of the weather and traffic data ingestion to additional countries beyond the current Australian pilot, followed by (2) integration of these environmental covariates (temperature, humidity, rainfall) and mobility indicators (traffic congestion, aviation passenger volumes) as exogenous regressors in forecasting and anomaly detection models—both extensions are at the design stage and the data are NOT yet incorporated as model inputs; (3) integration of mechanistic transmission models such as the susceptible–exposed–infectious–recovered (SEIR) compartmental framework [30] for cross-border spread modelling; (4) development of ensemble forecasting combining Prophet, autoregressive integrated moving average (ARIMA) [31], and deep learning approaches; (5) deployment of real-time alerting based on anomaly detection and R_t threshold breaches; and (6) pilot deployment with state-level public health agencies to validate the platform in operational surveillance workflows.

## Conclusions

EpiGIS Pro is an AI-powered geospatial intelligence platform that consolidates automated disease event extraction, environmental monitoring, human mobility data, and machine learning-based predictive analytics within a unified data infrastructure for global disease surveillance. The platform demonstrates that LLM-based extraction can achieve high-quality structured intelligence from heterogeneous news sources (92.4% success rate, 100% core field completeness), that hemisphere-concordant seasonal patterns can be reliably identified from WHO FluNet data across all evaluated countries, and that multiplicative Prophet forecasting can reduce RMSE by 21–29% relative to seasonal naive baselines when sufficient data volume is available (as demonstrated for the United States), while consistently outperforming log-transformed approaches across all evaluated settings. The modular architecture, reproducible evaluation framework, and 327,000 + integrated records across six data domains position EpiGIS Pro as both a practical surveillance tool and a research platform for advancing computational epidemiology methods.

## Electronic Supplementary Material

Below is the link to the electronic supplementary material.


Supplementary material 1.


## Data Availability

The source code for EpiGIS Pro is available from the corresponding author on reasonable request. WHO FluNet data are publicly available at https://www.who.int/tools/flunet. Disease event articles were collected from publicly accessible news APIs (NewsAPI.org, GNews, Google News RSS), WHO Disease Outbreak News, Reddit, and China CDC weekly bulletins. Weather data were obtained from OpenWeatherMap and Open-Meteo. Aviation route data were sourced from AviationStack and OpenSky Network. Traffic data were obtained from HERE Traffic API. Google Trends data are publicly accessible at https://trends.google.com. All third-party API data are subject to their respective terms of service.
