# Supplementary material for: EpiGIS pro: an AI-powered geospatial intelligence platform for integrated disease surveillance and predictive analytics
Source: Int J Health Geogr. 2026 Jun 16;25:45. doi: 10.1186/s12942-026-00479-1 (PMC13531970; doi:10.1186/s12942-026-00479-1)
Supplement: Supplementary file 1 [file 12942_2026_479_MOESM1_ESM.docx]

## Additional File 1: Supplementary Tables

**Table S1.** Disease type distribution for AI-extracted events (top 18 of 104 consolidated types, n = 326 processed events).

| Rank | Disease Type | Count | Percentage |
| --- | --- | --- | --- |
| 1 | Measles | 79 | 24.2% |
| 2 | COVID-19 | 15 | 4.6% |
| 3 | Avian Influenza (incl. A/H5N1) | 12 | 3.7% |
| 4 | Chikungunya | 8 | 2.5% |
| 5 | Nipah virus | 7 | 2.1% |
| 6 | Salmonella | 6 | 1.8% |
| 7 | Hepatitis B | 5 | 1.5% |
| 8 | Mpox | 5 | 1.5% |
| 9 | Foot-and-mouth disease | 4 | 1.2% |
| 10 | Polio | 4 | 1.2% |
| 11 | Cholera | 4 | 1.2% |
| 12 | Pertussis | 4 | 1.2% |
| 13 | Mumps | 4 | 1.2% |
| 14 | Leptospirosis | 4 | 1.2% |
| 15 | Anthrax | 4 | 1.2% |
| 16 | Rabies | 4 | 1.2% |
| 17 | Dysentery | 3 | 0.9% |
| 18 | Tuberculosis | 3 | 0.9% |
|  | Other (86 types) | 151 | 46.3% |

*Note: Percentages may not sum to exactly 100% due to rounding.*

**Table S2.** Complete forecast performance metrics for all 4 countries across three models and three horizons. Bold indicates best RMSE per country-horizon. SS = skill score relative to seasonal naive.

| Country | Horizon | Model | RMSE | MAE | MAPE (%) | sMAPE (%) | R² | SS |
| --- | --- | --- | --- | --- | --- | --- | --- | --- |
| **Australia** | 4 wk | Seasonal Naive | **113.5** | 103.8 | 66.1 | 113.0 | −15.20 | — |
|  | 4 wk | Prophet Multi | 147.2 | 142.8 | 76.9 | 126.4 | −20.44 | −0.30 |
|  | 4 wk | Prophet Log | 174.0 | 169.5 | 211.8 | 102.0 | −167.04 | −0.53 |
|  | 8 wk | Seasonal Naive | **114.1** | 105.0 | 66.7 | 115.9 | −13.86 | — |
|  | 8 wk | Prophet Multi | 143.0 | 135.7 | 73.2 | 124.8 | −14.40 | −0.25 |
|  | 8 wk | Prophet Log | 261.1 | 234.6 | 243.5 | 107.5 | −373.33 | −1.29 |
|  | 12 wk | Seasonal Naive | **130.1** | 116.3 | 68.0 | 117.7 | −7.33 | — |
|  | 12 wk | Prophet Multi | 164.9 | 151.2 | 74.6 | 122.8 | −8.53 | −0.27 |
|  | 12 wk | Prophet Log | 324.9 | 282.4 | 273.7 | 110.2 | −584.94 | −1.50 |
| **United States** | 4 wk | Seasonal Naive | 1891.7 | 1618.5 | 80.2 | 98.7 | −2.15 | — |
|  | 4 wk | Prophet Multi | **1344.1** | 1132.4 | 72.9 | 87.5 | −0.50 | 0.29 |
|  | 4 wk | Prophet Log | 2314.9 | 1924.7 | 159.1 | 93.2 | −3.45 | −0.22 |
|  | 8 wk | Seasonal Naive | 1637.9 | 1401.2 | 78.5 | 96.3 | −1.23 | — |
|  | 8 wk | Prophet Multi | **1286.8** | 1094.5 | 70.1 | 85.8 | −0.38 | 0.21 |
|  | 8 wk | Prophet Log | 2657.9 | 2215.6 | 172.3 | 98.1 | −4.87 | −0.62 |
|  | 12 wk | Seasonal Naive | 1452.0 | 1245.8 | 76.8 | 94.5 | −0.85 | — |
|  | 12 wk | Prophet Multi | **1131.9** | 968.3 | 68.4 | 83.2 | −0.12 | 0.22 |
|  | 12 wk | Prophet Log | 3608.2 | 3012.4 | 198.6 | 105.7 | −10.42 | −1.49 |
| **Japan** | 4 wk | Seasonal Naive | **31.2** | 25.8 | 55.3 | 78.9 | −8.42 | — |
|  | 4 wk | Prophet Multi | 50.2 | 43.1 | 68.7 | 95.2 | −24.11 | −0.61 |
|  | 4 wk | Prophet Log | 62.9 | 55.4 | 145.2 | 88.6 | −38.55 | −1.02 |
|  | 8 wk | Seasonal Naive | **39.6** | 33.2 | 58.1 | 82.4 | −5.67 | — |
|  | 8 wk | Prophet Multi | 49.9 | 42.8 | 65.9 | 93.1 | −9.22 | −0.26 |
|  | 8 wk | Prophet Log | 75.2 | 66.8 | 158.7 | 92.3 | −22.18 | −0.90 |
|  | 12 wk | Seasonal Naive | **54.9** | 46.5 | 61.2 | 85.7 | −3.12 | — |
|  | 12 wk | Prophet Multi | 61.1 | 52.4 | 64.8 | 91.8 | −4.05 | −0.11 |
|  | 12 wk | Prophet Log | 77.3 | 68.9 | 162.4 | 93.5 | −6.88 | −0.41 |
| **Germany** | 4 wk | Seasonal Naive | **8.8** | 6.2 | 42.5 | 65.3 | −12.88 | — |
|  | 4 wk | Prophet Multi | 22.1 | 18.5 | 85.4 | 112.3 | −88.15 | −1.51 |
|  | 4 wk | Prophet Log | 34.0 | 28.7 | 225.6 | 98.4 | −210.32 | −2.86 |
|  | 8 wk | Seasonal Naive | **24.0** | 18.8 | 55.2 | 78.6 | −4.25 | — |
|  | 8 wk | Prophet Multi | 29.9 | 24.2 | 72.8 | 98.5 | −7.12 | −0.25 |
|  | 8 wk | Prophet Log | 39.1 | 32.4 | 198.3 | 95.7 | −13.45 | −0.63 |
|  | 12 wk | Seasonal Naive | **33.6** | 26.8 | 58.7 | 82.1 | −2.85 | — |
|  | 12 wk | Prophet Multi | 38.7 | 31.5 | 68.5 | 95.2 | −3.88 | −0.15 |
|  | 12 wk | Prophet Log | 45.7 | 38.2 | 185.6 | 93.8 | −5.42 | −0.36 |

**Table S3.** Influenza seasonal profiles: mean weekly case counts and coefficient of variation (CV) by ISO week for 7 countries (WHO FluNet, 2019–2026, inf_all metric). Weeks with CV > 1.5 indicate high inter-annual variability.

| Country | Peak Week | Peak Mean Cases | Trough Week | Trough Mean Cases | Peak:Trough Ratio | Mean CV | Peak CV |
| --- | --- | --- | --- | --- | --- | --- | --- |
| Australia | 27 | 578.2 | 42 | 34.0 | 17.0 | 1.42 | 1.85 |
| Brazil | 18 | 124.5 | 36 | 19.5 | 6.4 | 1.18 | 1.62 |
| South Africa | 22 | 88.6 | 40 | 1.3 | 70.5 | 1.55 | 2.01 |
| United States | 1 | 4,852.3 | 32 | 99.6 | 48.7 | 1.35 | 1.78 |
| Japan | 52 | 312.8 | 30 | 11.6 | 27.1 | 1.41 | 1.92 |
| Germany | 5 | 91.4 | 35 | 0.2 | 457.3 | 1.68 | 2.15 |
| France | 4 | 68.2 | 33 | —† | —† | 1.52 | 1.95 |

*Peak Mean Cases and Trough Mean Cases represent the single week with the highest and lowest mean values, respectively. Peak:Trough Ratio is computed as the mean of the four highest weekly averages divided by the mean of the four lowest, and may therefore differ from single-week ratios. † France’s trough weeks contained zero values, precluding ratio computation.*
